# Inflammation in Muscle Repair, Aging, and Myopathies

**DOI:** 10.1155/2014/821950

**Published:** 2014-08-04

**Authors:** Marina Bouché, Pura Muñoz-Cánoves, Fabio Rossi, Dario Coletti

**Affiliations:** ^1^DAHFMO, Unit of Histology and Medical Embryology, Sapienza University of Rome, Via Antonio Scarpa 14, 00161 Rome, Italy; ^2^Cell Biology Group, Department of Experimental and Health Sciences, Pompeu Fabra University (UPF), CIBER on Neurodegenerative Diseases (CIBERNED), Institució Catalana de Recerca i Estudis Avançats (ICREA), Doctor Aiguader 88, 08003 Barcelona, Spain; ^3^The Biomedical Research Centre, UBC, Vancouver, BC, Canada V6T 1Z3; ^4^B2A Biology of Adaptation and Aging, Pierre and Marie Curie University (Paris 6), 7 quai St Bernard, 75252 Paris Cedex 5, France

Numerous recent studies have expanded our knowledge on the complexity of the immune system and its contribution to skeletal muscle repair, aging, and myopathies. Indeed, it is becoming clear that a precisely regulated cross talk between muscle and immune cells, involving endocrine/paracrine and cell-cell contact interactions, is required for muscle repair and maintenance of muscle homeostasis. Alterations of these mechanisms lead to unsuccessful repair in response to direct mechanical trauma (acute injury) or following secondary damage as a consequence of aging or genetic neuromuscular defects. In fact, though the capacity of muscle to regenerate relies primarily on a specific population of muscle stem cells, named satellite cells, the inflammatory cells that infiltrate the injured muscle appear to be as critical for successful regeneration. Conversely, if damage persists, as in chronic myopathies, inflammatory cell infiltration is perpetuated and leads to progressive muscle fibrosis, thus exacerbating disease severity.

While the number of scientific publications on the topic of skeletal muscle inflammation has steadily grown over the last two decades, the notion of inflammation as a common feature in muscle degeneration occurring in aging and myopathies and its association with altered muscle has to our knowledge never previously been addressed and discussed in dedicated journal issues before.

The main focus of this special issue is to bring together studies that used different experimental approaches* in vivo* or* in vitro* to dissect the dynamic changes taking place in specific immune cell populations, their cross talk with other cell types within the muscle milieu, and their contribution to normal versus pathological muscle repair.

Among the seven review articles, E. Rigamonti et al. discuss the current available literature about the role of macrophage populations in tissue injury and repair with a particular focus on skeletal muscle regeneration. Beyond their role in innate immunity, macrophages are now recognized as crucial players in orchestrating the healing of various injured tissues, including skeletal muscle. In this review the authors discuss the involvement of specific macrophage subpopulations in these processes and the complexity of defining macrophage subpopulations* in vivo*, since the* in vitro* criteria used to define M1 and M2 macrophages cannot be strictly applied to* in vivo* settings. Indeed, it is still a matter of debate whether the sequential presence of different macrophage populations results from a dynamic shift in macrophage polarization or from the recruitment of new circulating monocytes. Thus, to identify the molecular determinants of macrophage polarization is certainly one of the major tasks in developing effective targeted therapies for genetic defects of the tissue and muscle diseases associated with chronic inflammation.

However, although macrophages are emerging as indispensable for damage control and tissue remodeling following muscle injury and as principal mediators of pathological skeletal remodeling in diseases such as idiopathic inflammatory myositis (IIMs) and muscular dystrophies (MDs), the involvement of other immune cells in promoting or preventing muscle damage resolution is also emerging. The current knowledge and recent advances on the involvement of different innate immune cells in MDs and the emerging role of additional cell populations within the acquired immune response are being discussed in the review article by L. Madaro and M. Bouché.

It is widely believed that any disruption in the coordinated initiation, progression, and resolution of inflammation can lead to persistent muscle damage and impairment of regeneration, which in many cases is also characterized by development of fibrosis, as observed in MDs. Indeed, the process of fibrosis, whose underlying mechanisms are not fully elucidated yet, represents one important deleterious consequence of impaired muscle repair. In this special issue, Y. Kharraz et al. discuss new developments in our understanding of the mechanisms leading to fibrosis in Duchenne muscular dystrophy (DMD) and several recent advances towards reverting it, as well as potential treatments to attenuate disease progression.

Although blunting inflammation would not restore the primary defect in MDs, the emerging consensus is that multiple strategies addressing different aspects of the pathology, which may eventually converge, may be successful. Indeed, various therapies under development are directed toward rescuing the dystrophic muscle damage using gene transfer or cell-therapy.

A. Farini et al. discuss current knowledge about involvement of immune system responses to experimental therapies in MDs and how the different components of the immune system response are differently activated against cell- or gene-therapy both in a specific manner, linked to the type of cell or vector used, and according to MDs-specific immune-pathogenetic mechanisms.

In this context, S. M. Maffioletti et al. give an in-depth overview of the main players and issues involved in the mechanisms and dynamics that impair the efficacy of cell transplants in DMD and discuss potential approaches that might be beneficial for future regenerative therapies of skeletal muscle.

Muscle wasting linked to immune response is not, however, a feature restricted to genetic diseases.

In cancer cachexia, for example, inflammatory cytokines are important not only to establish tumor-host interaction and deregulate inflammatory response to tumor burden, but also to mediate muscle wasting by directly targeting muscle tissue. In the review by J. K. Onesti and D. C. Guttridge a summary of the clinical implications, background of inflammatory cytokines, and the origin and sources of procachectic factors is provided. Molecular mechanisms and pathways are also described to elucidate the link between the immune response caused by the presence of the tumor and cytokine-dependent inhibition of muscle regeneration, ultimately resulting in muscle wasting.

Muscle wasting is also a feature of IIMs, a wide range of autoimmune diseases, characterized clinically by reduced muscle endurance and weakness, chronic inflammation, and infiltration by immune/inflammatory cells in skeletal muscles. Treatments for IIMs are based on lifelong immunosuppressive therapy, which comes with well-known adverse effects; recovery is incomplete for many patients. More effective therapies, with reduced side effects, are highly desirable. In this context, C. Crescioli proposes vitamin D receptor (VDR) agonists as candidates in future treatment of IIMs. In her review she summarizes the pleiotropic anti-inflammatory properties of VDRs with potentially limited adverse effects.

Similarly, A. Costa et al., in one out of the four original contributions in this issue, propose Arg-vasopressin- (AVP-) dependent pathways as an interesting strategy to counteract muscle decline in aging or myopathies. Indeed, the authors show that overexpression of the AVP receptor V1a in skeletal muscle* in vivo* increases the expression of regenerative markers, modulates immune response, and attenuates fibrogenesis, thus enhancing muscle regeneration and counteracting the negative effects of the proinflammatory cytokine TNF.

On the other hand, M. Pelosi et al. addressed the effects of IL-6, a multifaceted pleiotropic cytokine, on the myogenic program. In physiological conditions IL-6 is required for muscle homeostasis, but in several pathological conditions its level increases and it contributes to muscle wasting. In this paper the authors explored the molecular mechanisms underlying IL-6-dependent inhibition of the myogenic program and identified potential molecular mediators of these effects.

It is well known that the inflammatory response plays an important role also in the development of ischemic heart disease. In this context, G. D. Duerr et al. demonstrate in this issue that osteopontin (OPN) has a cardioprotective effect. OPN is a matricellular protein and cytokine involved in the regulation of macrophage function and as a remodeling-associated mediator in different tissues. Using* in vivo* models they show that lack of OPN prevents heart remodeling in a murine model of ischemic cardiopathy, altering the expression of contractile elements and chemokines, as well as remodeling factors. These findings may further support the therapeutical perspective for osteopontin as a possible target in protection of the ischemic heart.

To conclude this special issue, C. Sciorati et al. characterize the ability of the 7-Tesla magnetic resonance imaging (MRI) to reveal specific inflammatory events in the skeletal muscle using a mouse model of IIM. One of the limits in understanding the etiopathogenesis of muscle inflammatory diseases is the paucity of approaches for the noninvasive study of inflamed tissues. The authors provide an in-depth noninvasive characterization of this myositis model, proving the efficacy of MRI as an informative and noninvasive analytical tool for studying* in vivo* immune-mediated muscle involvement. Indeed, the availability of noninvasive tools to monitor muscle inflammation will permit reduction in the number of animals necessary for experimental studies and will increase the amount of relevant information that can be derived from single animals followed over time. At the same time, this imaging modality will complement information derived from histopathological studies that encompass only limited areas of tissue and therefore suffer from potential sampling bias.

What emerges from this special issue is that inflammation-regulated muscle repair in aging and disease is an active and rapidly advancing research field, and we hope that the papers here collected will help to connect fundamental science with biomedical research on severe myopathies, with the aim of opening new venues for therapy. It is our wish that these papers will also be a useful tool for the inflammation and skeletal muscle research community and that they will attract and motivate investigators from different scientific areas to this important field of research.

